# Simple Clinical Metrics Enhance AFP to Effectively Identify Cirrhotic Patients With Complicating Hepatocellular Carcinoma at Various AFP Levels

**DOI:** 10.3389/fonc.2019.01478

**Published:** 2020-01-24

**Authors:** Xi Zhang, Ting Wang, Kun-He Zhang, Si-Hai Chen, Yu-Ting He, Yu-Qi Wang

**Affiliations:** ^1^Center for Experimental Medicine Research, The First Affiliated Hospital of Nanchang University, Nanchang, China; ^2^Department of Gastroenterology, Jiangxi Institute of Gastroenterology and Hepatology, The First Affiliated Hospital of Nanchang University, Nanchang, China

**Keywords:** hepatocellular carcinoma, liver cirrhosis, diagnostic model, alpha-fetoprotein, clinical metrics, calibration curve analysis, decision curve analysis

## Abstract

**Background:** Hepatocellular carcinoma (HCC) frequently occurs in cirrhosis and closely relates to poor prognosis of cirrhotic patients. Alpha-fetoprotein (AFP) is the most widely used biomarker in HCC diagnosis but not sensitive and specific to detect HCC at low AFP levels. In order to enhance the ability of AFP to detect HCC developed on cirrhosis, we attempted to combine AFP with conventional clinical metrics to develop a simple and effective method for identifying cirrhotic patients with complicating HCC at various AFP levels.

**Methods:** Cirrhotic patients with or without HCC hospitalized to receive therapy for the first time were recruited and their clinical data were retrospectively collected. A model for diagnosing HCC was developed with routine clinical metrics and AFP by binary logistic regression analysis and internally validated. The goodness of fit, diagnostic accuracy and clinical usefulness of the model were evaluated using a calibration curve, the area under the receiver operating characteristic curve (AUROC) and a decision curve analysis, respectively.

**Results:** A total of 574 patients with cirrhosis mainly caused by hepatitis B were recruited in this study, including 286 cases of simple cirrhosis (LC) and 288 cases of cirrhosis with HCC (LCC) (124 AFP-negative), with an average age of 53.2 ± 12.1 years and 81.4% males. Twelve of the 19 clinical metrics (age, gender, AFP, liver function tests, serum electrolytes, and coagulation tests) significantly differed between the LC and LCC groups. A model was successfully developed with age, AFP, Na^+^, Cl^−^, alkaline phosphatase, and activated partial thromboplastin time, which exhibited good performance in diagnosing LCC, with an AUROC of 0.918 (95%CI 0.895–0.940), 82.3% sensitivity, 89.5% specificity, and 85.9% accuracy for all patients, which were much higher values than those for AFP [0.846 (95%CI 0.815–0.878), 72.9, 81.5, and 77.2%, respectively]. For cirrhotic patients complicated with AFP-negative HCC, the model showed an AUROC of 0.854 (95%CI 0.812–0.896), 68.5% sensitivity, 86.6% specificity, and 80.0% accuracy. A high net benefit could be obtained in clinical decision making according to the model.

**Conclusion:** A diagnostic model combining simple clinical metrics with AFP is valuable for the identification of cirrhotic patients complicating HCC with various AFP levels.

## Introduction

Liver cancer, mainly hepatocellular carcinoma (HCC), was estimated to be the sixth most frequent cancer and the fourth cause of cancer death worldwide in 2018, with appropriately 841,000 new cases and 782,000 deaths annually (1); additionally, the incidence of HCC has been increasing in the past two decades and is expected to increase until 2030 in some countries, including the United States. HCC is a frequent complication of liver cirrhosis (LC). Approximately 70–90% of HCC occurs on a background of cirrhosis (2), and in cirrhotic patients, the 5-year cumulative risk of developing HCC is 17% in East Asia and 10% in Western Europe and the United States (3). Consistent screening of HCC development could increase the survival of patients with cirrhosis, but it has been underused (4). Therefore, the surveillance of HCC development is a key issue in the management of cirrhotic patients.

According to the 2018 Practice Guidance by the American Association for the Study of Liver Diseases (5), the modality recommended for HCC surveillance in cirrhotic patients is ultrasound, with or without alpha-fetoprotein (AFP), every 6 months, indicating that AFP is not as important as ultrasound for HCC surveillance. However, the surveillance strategy of combining AFP and ultrasound examinations at 6-month intervals is estimated to detect more than triple the number of patients with operable HCC tumors at the time of diagnosis and reduce almost half the number of deaths from HCC compared with no surveillance (6). More interestingly, AFP is more important than ultrasound, as this test can identify approximately six times as many small tumors as ultrasound in the surveillance. Furthermore, the harms of surveillance, which are mostly related to false positives and indeterminate tests, are more often associated with ultrasound when compared with AFP (7). Hence, AFP should not be ignored in HCC detection.

AFP has been the most widely used serum biomarker for the diagnosis of HCC due to its simplicity, non-invasiveness and good repeatability, but this test is not sensitive (8), especially in small and early-stage HCC. Accordingly, tremendous efforts have been made in the biomarker investigation of liver cancer in the past decades, and many new biomarkers have been reported. However, few new biomarkers have been demonstrated to be valuable in clinical practice. The most reasonable strategy to improve diagnostic performance is the combination of AFP with other biomarkers of HCC. The combination of AFP with des-gamma-carboxyprothrombin (DCP), another clinically used biomarker of HCC, could increase the sensitivity (9), but the improvement is insufficient, especially for early stage HCC (10).

Conventional laboratory tests are of value in the prediction of HCC development in cirrhotic patients. The algorithm based on levels of AFP, platelets, and alanine aminotransaminase (ALT), along with age, could predict whether HCC is likely to develop within 6 months in patients with hepatitis C virus-associated cirrhosis (11). In newly developed cirrhosis associated with hepatitis B virus (HBV), the levels of ALT, AFP, and HBV markers as well as age were predictors for HCC development (12). In our previous work, we found that AFP combined with serum fluorescence intensity and conventional laboratory tests was valuable in the diagnosis of primary hepatic carcinoma (13, 14). The combination of clinical data with AFP may be a simple and practicable method for the identification of cirrhotic patients complicated with HCC.

In the present study, we attempted to develop and comprehensively evaluate an algorithm based on the combination of AFP and clinical metrics to simply and practically diagnose cirrhotic patients complicated with HCC at various AFP levels.

## Patients and Methods

### Collection of Patients and Related Clinical Data

**Figure 1:**
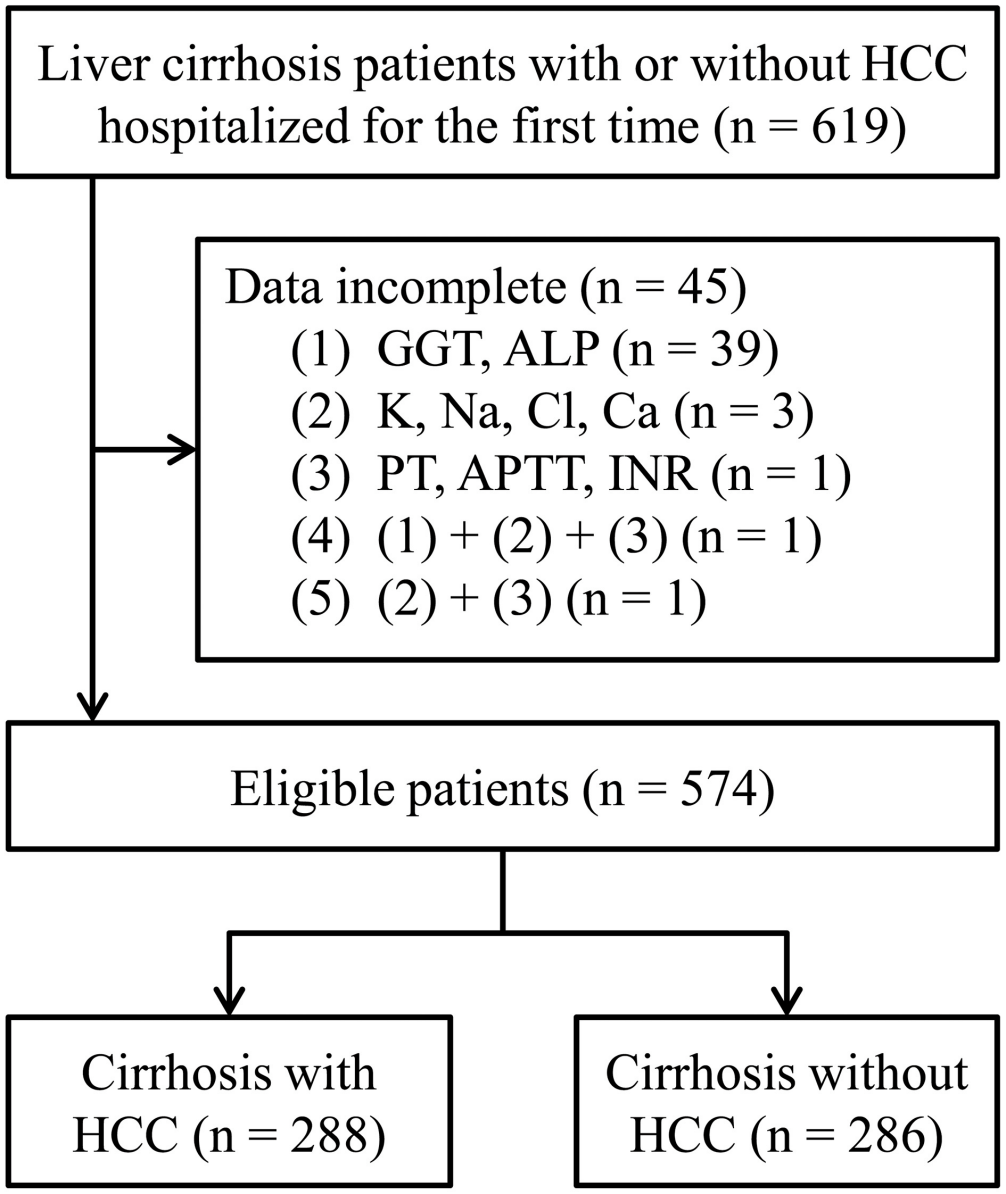
The flowchart of patient selection. HCC, hepatocellular carcinoma; GGT, gamma-glutamyl transferase; ALP, alkaline phosphatase; PT, prothrombin time; APTT, activated partial thromboplastin time; INR, international standardized ratio.

Cirrhotic patients hospitalized in the First Affiliated Hospital of Nanchang University receiving therapy for the first time were retrospectively collected, and those previously treated with special local therapies or those with incomplete clinical data were excluded. The cirrhosis was determined by the evidence of cirrhotic liver and the presence of gastroesophageal varices on B-ultrasound, CT and/or MRI examinations in the presence of chronic liver disease. HCC patients were diagnosed by histological examination or by imaging characteristics as defined in the guideline (15). The flowchart of patient selection is shown in Figure 1. Available clinical data, including demographic information, etiologic data, laboratory test results of liver function, AFP, electrolytes and coagulation function, and results of medical imaging, were collected from the medical records of the patients.

### Statistical Analyses of the Clinical Data

The patients were assigned to two groups: simple liver cirrhosis (LC) and cirrhosis complicated with HCC (LCC). The Child-Pugh score and grade of each patient were determined. The difference in clinical metrics between the two groups was compared using a *t*-test or Pearson's Chi-squared test. The area under the receiver operating characteristic (ROC) curve (AUROC) was utilized to evaluate the diagnostic value of each clinical metric. *P* < 0.05 was considered significant. All data analyses were performed using SPSS version 23 for Windows (IBM, NY, USA).

### Development and Evaluation of a Diagnostic Model for Cirrhosis With HCC

Because a skewed distribution existed in most clinical metrics, we transformed the metrics by natural logarithm. The patients were randomly divided into a training set and a validation set. A diagnostic model was established using binary logistic stepwise regression analysis based on the training set and validated with the validation set. The fit-goodness of the model was determined by the Hosmer-Lemeshow test and a calibration curve. The diagnostic power of the model was evaluated using AUROC and sensitivity, specificity, accuracy, positive/negative predictive values, and the positive/negative likelihood ratio. The clinical usefulness of the model was evaluated by decision curve analysis (16). The calibration curve and decision curve were plotted using R software.

## Results

### Demographic and Clinical Data for the Patients

A total of 574 patients with cirrhosis, including 288 LCC (124 AFP-negative) and 286 LC patients, were recruited into the present study. The demographic and clinical data for these patients are shown in Table 1.

**Table 1:** Demographic and clinical characteristics of the patients.

	**LC (*n* = 286)**	**LCC (*n* = 288)**	***P***
Age (mean ± SD, years)	50.7 ± 12.2	55.7 ± 11.4	<0.001^a^
Gender [*n* (%)]			
Male	229 (80.1)	238 (82.6)	0.454^b^
Female	57 (19.9)	50 (17.4)	
Etiology [*n* (%)]			
HBV	229 (80.1)	230 (79.9)	<0.001^b^
Other^†^	7 (2.4)	15 (5.2)	
Mixed	41(14.3)	15 (5.2)	
Unknown	9 (3.1)	28 (9.7)	
Laboratory blood tests (mean ± SD)			
AFP (μg/L)	10.9 ± 49.9	417.4 ± 551.6	<0.001^a^
ALT (U/L)	56.3 ± 185.7	57.8 ± 100.3	0.901^a^
AST (U/L)	61.9 ± 119.9	88.6 ± 139.1	0.014^a^
TBIL (μmol/L)	35.6 ± 68.3	30.0 ± 38.1	0.230^a^
DBIL (μmol/L)	20.5 ± 43.0	17.3 ± 28.1	0.281^a^
TP (g/L)	61.0 ± 8.7	64.6 ± 7.2	<0.001^a^
ALB (g/L)	32.1 ± 5.7	35.0 ± 6.5	<0.001^a^
GLB (g/L)	28.9 ± 7.3	29.6 ± 6.2	0.183^a^
GGT (U/L)	55.0 ± 85.5	122.1 ± 145.6	<0.001^a^
ALP (U/L)	104.6 ± 77.9	162.6 ± 132.2	<0.001^a^
K^+^ (mmol/L)	4.1 ± 0.6	4.1 ± 0.6	0.243^a^
Na^+^ (mmol /L)	138.4 ± 3.8	139.0 ± 3.8	0.048^a^
Cl^−^ (mmol /L)	106.2 ± 5.0	102.7 ± 4.5	<0.001^a^
Ca^2+^ (mmol /L)	2.1 ± 0.2	2.2 ± 0.2	<0.001^a^
PT (s)	14.2 ± 3.8	13.2 ± 2.3	<0.001^a^
APTT (s)	41.4 ± 12.4	36.5 ± 9.2	<0.001^a^
INR	1.2 ± 0.3	1.2 ± 0.2	0.056^a^
Child-Pugh grade [*n* (%)]			
A	154 (53.8)	160 (55.6)	0.029^b^
B	60 (21.0)	79 (27.4)	
C	72 (25.2)	49 (17.0)	

† *The other causes include hepatitis C, alcoholic liver disease, schistosomiasis*.

a *Student t-test*.

b *Pearson's Chi-squared test*.

*LCC, liver cirrhosis with hepatocellular carcinoma; LC, liver cirrhosis; HBV, hepatitis B virus; AFP, alpha-fetoprotein; ALT, alanine aminotransaminase; AST, aspartate aminotransaminase; TBIL, total serum bilirubin; DBIL, direct serum bilirubin; TP, total serum protein; ALB, serum albumin; GLB, serum gamma-globulin; GGT, gamma-glutamyl transferase; ALP, alkaline phosphatase; PT, prothrombin time; APTT, activated partial thromboplastin time; INR, international standardized ratio*.

### The Diagnostic Value of Single Metrics for Cirrhosis Complicated With HCC

A total of 19 metrics of demographic and clinical data were evaluated for LCC diagnosis by the ROC curve. Twelve of the 19 metrics exhibited significant diagnostic value for LCC with either AFP <400 ng/mL or AFP at all levels (Table 2). The AUROCs of single metrics were generally insufficient for diagnosing LCC.

**Table 2:** AUROCs of single metrics for the diagnosis of cirrhosis complicated with hepatocellular carcinoma.

**Metrics**	**AUROC (95% CI)**	
	**Patients with AFP <400 ng/mL**	**Patients with all AFP levels**
AFP	0.764 (0.720–0.808)^**^	0.846 (0.815–0.878)^**^
Age	0.666 (0.617–0.715)^**^	0.626 (0.580–0.671)^**^
Gender	0.514 (0.460–0.567)	0.513 (0.466–0.560)
ALT	0.627 (0.576–0.679)^**^	0.640 (0.594–0.685)^**^
AST	0.636 (0.585–0.686)^**^	0.662 (0.618–0.706)^**^
TBIL	0.501 (0.448–0.554)	0.503 (0.456–0.550)
DBIL	0.556 (0.502–0.611)^*^	0.551 (0.504–0.599)^*^
TP	0.616 (0.564–0.667)^**^	0.620 (0.574–0.665)^**^
ALB	0.606 (0.553–0.659)^**^	0.628 (0.583–0.673)^**^
GLB	0.555 (0.503–0.607)^*^	0.543 (0.495–0.590)
GGT	0.701 (0.654–0.749)^**^	0.733 (0.692–0.773)^**^
ALP	0.703 (0.655–0.750)^**^	0.701 (0.658–0.743)^**^
K^+^	0.553 (0.500–0.606)	0.533 (0.486–0.580)
Na^+^	0.554 (0.500–0.609)^*^	0.561 (0.514–0.608)^*^
Cl^−^	0.726 (0.680–0.772)^**^	0.734 (0.693–0.775)^**^
Ca^2+^	0.722 (0.675–0.769)^**^	0.734 (0.694–0.774)^**^
PT	0.597 (0.545–0.649)^**^	0.581 (0.534–0.627)^**^
APTT	0.647 (0.597–0.697)^**^	0.630 (0.584–0.675)^**^
INR	0.542 (0.489–0.595)	0.525 (0.477–0.572)

* P <0.05 and

** *P <0.01. AUROC, area under the receiver operating characteristic curve; CI, interval confidence; AFP, alpha-fetoprotein; ALT, alanine aminotransaminase; AST, aspartate aminotransaminase; TBIL, total serum bilirubin; DBIL, direct serum bilirubin; TP, total serum protein; ALB, serum albumin; GLB, serum gamma-globulin; GGT, gamma-glutamyl transferase; ALP, alkaline phosphatase; PT, prothrombin time; APTT, activated partial thromboplastin time; INR, international standardized ratio*.

### The Diagnostic Performance of the Model for Cirrhosis Complicated With HCC

**Figure 2:**
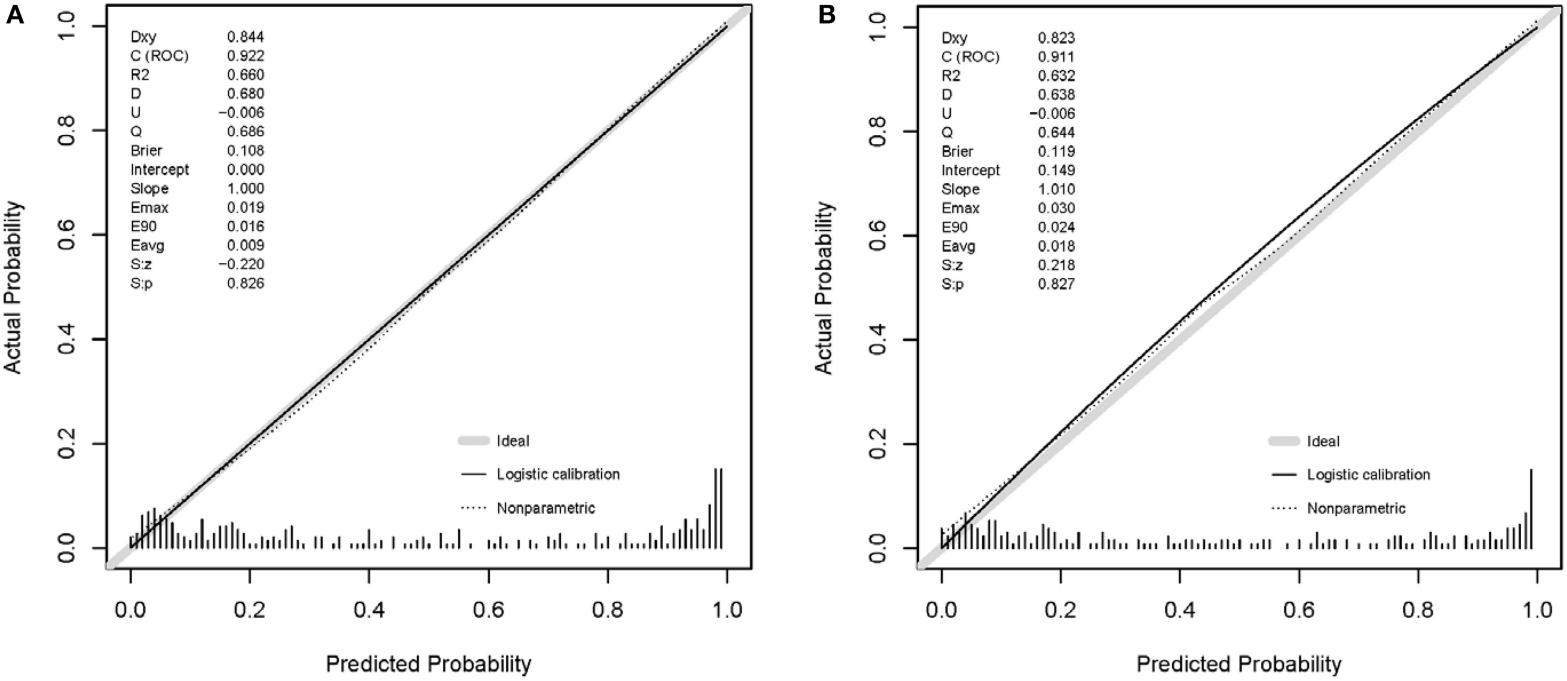
Calibration curves of the model. **(A)** Training set; **(B)** validation set.

**Figure 3:**
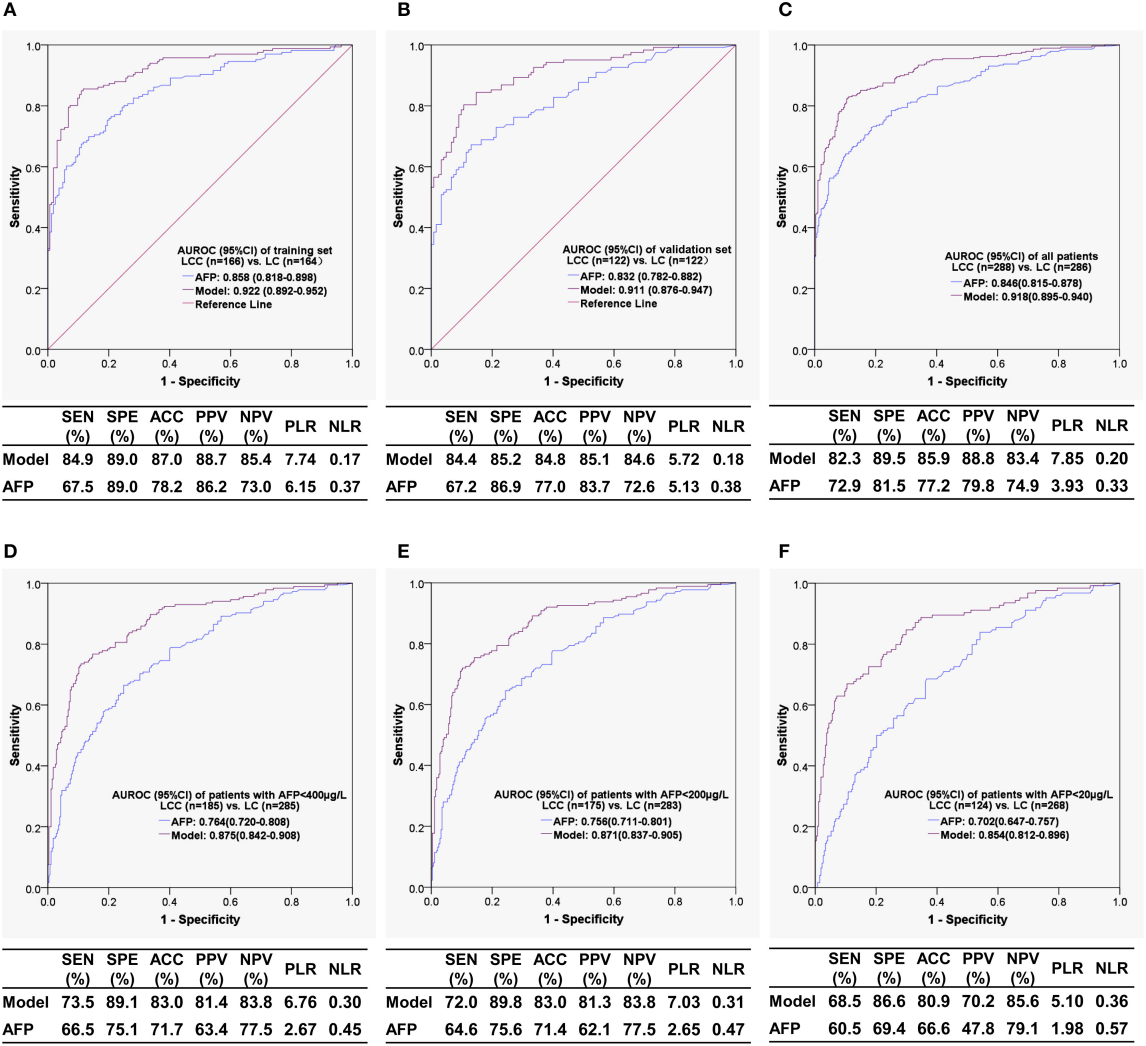
The receiver operating characteristic curves and diagnostic performances of the model for the diagnosis of liver cirrhosis complicated with hepatocellular carcinoma. **(A)** Training set; **(B)** validation set; **(C)** all patient set; **(D)** the set of patients with AFP < 400 μg/L; **(E)** the set of patients with AFP < 200 μg/L; **(F)** the set of patients with AFP < 20 μg/L. AUROC, area under the receiver operating characteristic curve; CI, confidence interval; LCC, liver cirrhosis complicated with hepatocellular carcinoma; LC, liver cirrhosis; AFP, alpha-fetoprotein; SEN, sensitivity; SPE, specificity; ACC, accuracy; PPV, positive predictive value; NPV, negative predictive value; PLR, positive likelihood ratio; NLR, negative likelihood ratio.

The patients were randomly divided into a training set (LC *n* = 164, LCC *n* = 166) and a validation set (LC *n* =122, LCC *n* = 122) at an appropriate ratio of 6:4. Based on the training set, a diagnostic model to identify cirrhotic patients complicated with HCC was successfully developed with six variables (Table 3), which had a Nagelkerke *R*^2^ of 0.660 and a *p*-value of 0.718 in the Hosmer-Lemeshow test. The calibration curves showed that the predicted probability was well in accordance with the observed probability in the training set and validation set (Figure 2), with a very small maximum error (Emax) and an average error (Eevg) for the training set (0.019 and 0.009, respectively) and validation set (0.030 and 0.018, respectively). The model exhibited AUROCs from 0.922 to 0.854 and accuracies from 87.0 to 80.9% in various datasets, and all of these values were much higher than those obtained with AFP, especially with AFP <400 μg/L (Figure 3).

**Table 3:** Variables entered the diagnostic model for cirrhosis complicated with hepatocellular carcinoma.

**Variable**	**B**	**SE**	**Wald**	**P**	**OR (95% CI)**
LnAFP	0.762	0.100	58.196	<0.001	2.143 (1.762–2.607)
LnALP	0.821	0.323	6.463	0.011	2.272 (1.207–4.278)
LnAPTT	−2.257	0.730	9.550	0.002	0.105 (0.0254–0.438)
Cl^−^	−0.183	0.044	17.246	<0.001	0.833 (0.764–0.908)
Na^+^	0.151	0.053	8.021	0.005	1.163 (1.048–1.291)
Age	0.054	0.015	13.056	<0.001	1.055 (1.025–1.086)
Constant	−2.116	7.083	0.089	0.765	0.121

*OR, odds ratio; CI, confidence interval; Ln, natural logarithm transformed; AFP, alpha-fetoprotein; ALP, alkaline phosphatase; APTT, activated partial thromboplastin time*.

### The Usefulness of the Model in Clinical Decision Making

**Figure 4:**
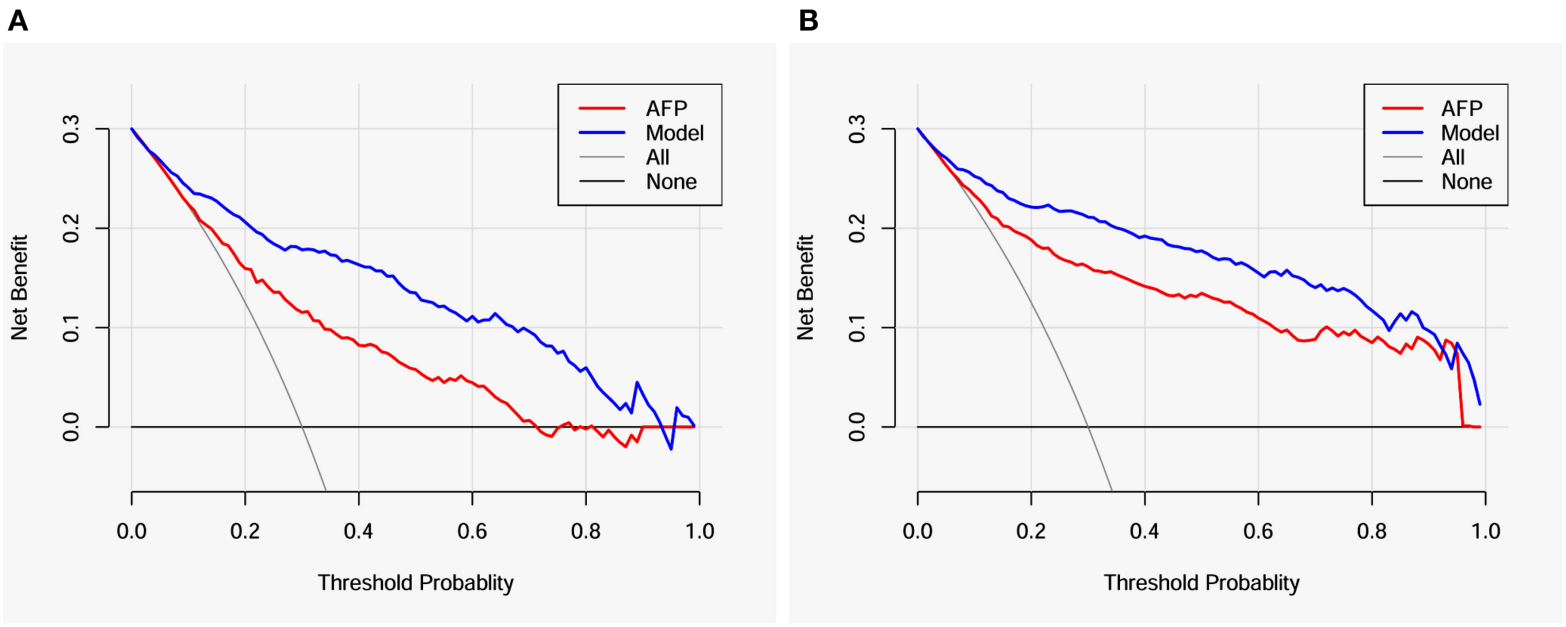
Decision curves for the model and AFP. **(A)** Patients with AFP < 400 μg/L; **(B)** all patients. AFP, alpha-fetoprotein.

The value of the model in clinical decision making for further diagnostic examinations to detect HCC in cirrhotic patients was evaluated by a decision curve analysis. When the threshold probabilities ranged from ~10 to 90% for patients with AFP < 400 μg/L (Figure 4A) and from ~10 to 95% for all patients (Figure 4B), much higher net benefits could be gained in the clinical decision on whether to take further diagnostic intervention according to the probability of complicating HCC provided by the model compared with non-selection (toward “all” or “none” patients). Compared with AFP, the model also exhibited higher net benefits in deciding whether further diagnostic intervention is needed for identifying HCC in cirrhotic patients. The detailed net benefits of the model in clinical decision making for patients with AFP < 400 μg/L are shown in Table 4.

**Table 4:** Comparison of the net benefits in clinical decisions regarding the diagnosis of hepatocellular carcinoma in cirrhotic patients with AFP < 400 μg/L.

**Pt (%)**	**Net benefit**		**Advantage of the model**	
	**Intervention to all patients**	**Intervention based on the model**	**Increment of net benefit**	**Unnecessary intervention avoided per 100 patients**
3	0.278	0.278	0.000	0
5	0.263	0.268	0.005	10
8	0.239	0.253	0.014	16
10	0.222	0.241	0.019	17
20	0.125	0.206	0.081	32
30	0.000	0.178	0.178	42
40	−0.167	0.163	0.330	50
50	−0.400	0.135	0.535	54
60	−0.750	0.111	0.861	57
70	−1.333	0.096	1.429	61
80	−2.500	0.060	2.560	64
90	−6.000	0.033	6.033	67
96	−16.50	0.019	16.519	69

*Pt, threshold probability; “Increment of net benefit” is calculated as: net benefit of the model – net benefit of intervention to all; “Unnecessary intervention avoided per 100 patients” is calculated as Vickers and Elkin (17): (net benefit of the model – net benefit of intervention to all)/(pt/(1– pt)) × 100*.

## Discussion

In the present study, a diagnostic model incorporating AFP and five clinical metrics was developed and internally validated to identify cirrhotic patients complicated with HCC. Compared with AFP, the model exhibited much higher diagnostic performances for cirrhotic patients complicated with HCC, particularly for patients with AFP < 400 μg/L. For AFP-negative HCC, the model was still well in diagnostic performance. Decision curve analysis proved the model to be valuable in clinical decision-making.

Our model had an AUROC of 0.918 for the diagnosis of HCC, which is better than or comparable to that for well-known serum HCC biomarkers alone and in combination with AFP, as shown in a meta-analysis (18), in which the summary ROCs were 0.79 and 0.87 for DCP, respectively, 0.71 and 0.83 for AFP-L3, respectively, 0.76 and 0.85 for glypican-3 (GPC3), respectively, and 0.91 and 0.93 for Golgi protein 73 (GP73), respectively. Our model also exhibited higher diagnostic performance compared with a similarly diagnostic model developed by Patel and Yopp (19), whose model incorporated six variables (male gender, Black race, viral etiology, alkaline phosphatase >1.5 times upper limit normal, FIB-4, and AFP > 20 ng/mL) to determine the presence of HCC in cirrhotic patients predominantly caused by hepatitis C and presented AUROCs of 0.84 and 0.83 in derivation and validation cohorts, respectively. We have not found similar diagnostic models developed with clinical parameters for identifying HCC in cirrhosis mainly caused by hepatitis B.

The diagnosis of HCC is not difficult in cirrhotic patients with AFP ≥ 400 μg/L, whereas the occurrence of HCC should be determined in patients with low-level or negative AFP concentrations. Our model exhibited a powerful ability to identify HCC in cirrhotic patients with AFP < 400 μg/L, with an AUROC of 0.875, sensitivity of 73.5%, specificity of 89.1%, and accuracy of 83.0%; more than 10% of diagnostic accuracy was obtained by the model compared with AFP, indicating that the conventional clinical indicators remarkably enhanced the ability of AFP to identify HCC. More importantly, our model also had high diagnostic performance in AFP-negative (<20 μg/L) cirrhotic patients with HCC, with an AUROC of 0.854, sensitivity of 68.5%, specificity of 86.6%, and accuracy of 80.9%, respectively, which was superior or comparable to typical HCC biomarkers for AFP-negative HCC diagnosis, such as DCP (AUROC = 0.73) (20), AFP-L3 (AUROC = 0.61) (21), GP73 (AUROC = 0.78) (21), GPC3 (AUROC = 0.641) (22), and Dickkopf-1 (AUROC = 0.834) (23).

In addition to age and AFP (two well-known variables valuable for HCC diagnosis), alkaline phosphatase (ALP), activated partial thromboplastin time (APTT), Cl^−^ and Na^+^ were also independent predictors in the model. Serum ALP levels were much higher in cirrhotic patients with HCC, although ALT, total serum bilirubin (TBIL) and direct serum bilirubin (DBIL) levels were similar in cirrhotic patients with or without HCC. ALP is a hydrolase enzyme primarily present in the liver, bile duct, bone, kidney, and placenta and is closely associated with the development of HCC. Higher serum ALP levels are found in HCC patients with larger tumor size (24) and extrahepatic metastasis (25). ALP is an independent prognostic factor for HCC patients (26) and one of the parameters integrated in some staging systems. The ratio of serum albumin (ALB) to ALP is a powerful index to the prognostication of HCC (27) and cholangiocarcinoma (28).

In univariate and multivariate analyses, APTT was a significant predictor for cirrhotic patients with HCC, with a shorter time compared with simple cirrhotic patients. APTT measures the effectiveness of the intrinsic coagulation pathway and is clinically associated with relevant hypercoagulability (29), but this variable usually does not reflect hepatic dysfunction (30). It is well-known that cancer patients are more prone to develop venous thrombosis. Indeed, HCC patients present with a significant risk of venous thrombosis, especially in the portal vein (31). Cirrhotic patients with HCC exhibit hypercoagulability, with a double incidence of portal vein thrombosis compared to simple cirrhotic patients (24.4 vs. 11.4%) (32). The underlying mechanism is that the rebalanced and unstable hemostatic status of liver cirrhosis can be easily tipped toward thrombotic complications by superimposed conditions, including HCC (33).

Serum electrolyte derangements are common in patients with decompensated cirrhosis. In the present study, serum sodium and chloride were the independent predictors of cirrhotic patients with complicating HCC, and we observed higher sodium levels and lower chloride levels in patients with HCC than in patients without HCC. However, we have not retrieved literature on the differences in serum electrolytes between cirrhotic patients with and without HCC and on the diagnostic significance for cirrhotic patients with complicating HCC, although hyponatremia is an independent prognostic predictor of HCC with cirrhosis (34). It is worth investigating the association of serum electrolyte derangements with HCC development in cirrhotic patients.

Although a powerful model was established and internally validated for the identification of patients complicated with HCC, at least three limitations existed in the present study. First, this study was a retrospective study with hospitalized patients, which may have caused selection bias due to a lack of outpatients. Second, the model was developed based on a patient cohort from a single center; the model should be validated by an external patient cohort. Third, hepatitis B was the principal cause of cirrhosis in the present study, and other common causes of cirrhosis, such as hepatitis C and alcohol abuse, accounted for very low proportions in the patient cohort. Hence, prospective multicenter studies are required to validate the model based on cohorts closer to the real-world composition of etiology and illness severity.

In summary, we successfully developed and internally validated a simple and robust model that was able to identify HCC with various AFP levels in cirrhotic patients, including AFP-negative HCC. The model incorporated AFP and five routine clinical parameters that are readily available and exhibited high net benefit in clinical decision-making, suggesting a good complement to AFP and high feasibility in HCC detection. With external validation, the model may be valuable in the diagnosis and surveillance of HCC development in cirrhotic patients.

## Data Availability Statement

The datasets generated for this study are available on request to the corresponding author.

## Ethics Statement

The studies involving human participants were reviewed and approved by the Ethical Committee of the First Affiliated Hospital of Nanchang University. Written informed consent for participation was not required for this study in accordance with the national legislation and the institutional requirements.

## Author Contributions

KZ and XZ conceived and designed the study. KZ, XZ, and TW analyzed and interpreted the data. YH, SC, and YW collected clinical data. KZ and XZ prepared and revised the manuscript. All authors have read and approved the contents of the final manuscript.

### Conflict of Interest

The authors declare that the research was conducted in the absence of any commercial or financial relationships that could be construed as a potential conflict of interest.
